# Factors associated with the implementation of programs for drug abuse prevention in schools

**DOI:** 10.1590/S1518-8787.2016050005819

**Published:** 2016-07-26

**Authors:** Ana Paula Dias Pereira, Ângela Tavares Paes, Zila M Sanchez

**Affiliations:** I Programa de Pós-Graduação em Saúde Coletiva. Universidade Federal de São Paulo. São Paulo, SP, Brasil; II Setor de Estatística Aplicada. Pró-reitoria de Pós-Graduação e Pesquisa. Universidade Federal de São Paulo. São Paulo, SP, Brasil; III Departamento de Medicina Preventiva. Centro Brasileiro de Informações Sobre Drogas Psicotrópicas. Universidade Federal de São Paulo. São Paulo, SP, Brasil

**Keywords:** School Health, Adolescent Behavior, Street Drugs, Substance-Related Disorders, prevention & control, Program Evaluation, Health Promotion

## Abstract

**OBJECTIVE:**

To analyze if characteristics of managers, schools, and curriculum are associated with the implementation of programs for drug abuse prevention in elementary and high schools.

**METHODS:**

Cross-sectional study, with random sample of 263 school managers. Data were collected between 2012 and 2013 by a program that sends forms via internet. A closed self-filling questionnaire was applied online. Statistical analysis included Chi-square tests and logistic regression models. The outcome variable was the presence of program for drug abuse prevention inserted in the daily life and educational program of the school. The explanatory variables were divided into: demographic data of the manager; characteristics of the school and of the curriculum; health education; and drug use in the school.

**RESULTS:**

We found that 42.5% (95%CI 36.1–49.1) of the evaluated schools had programs for drug abuse prevention. With the multiple logistic regression model, we observed that the more time the manager has worked with education, the chance of the school having a program increased at about 4.0%. Experimenting with innovative teaching techniques also increased at about six times the chance of the school developing a program for drug abuse prevention. The difficulties in the implementation of the programs were more present in state and municipal schools, when compared with private schools, due to, for instance: lack of teaching materials, lack of money, and competing demands for teaching other subjects.

**CONCLUSIONS:**

The implementation of programs for drug abuse prevention in the city of Sao Paulo is associated with the experience of the manager in education and with the teaching strategies of the school.

## RESUMO

**OBJETIVO:**

Analisar se características dos dirigentes, das escolas e do currículo escolar estão associadas à implantação de programas de prevenção ao uso de drogas nas escolas do ciclo fundamental II e médio.

**MÉTODOS:**

Estudo transversal, com amostra aleatória sistemática de 263 dirigentes escolares. Os dados foram coletados nos anos de 2012 e 2013 por meio de um programa de envio de formulários pela internet. Aplicou-se questionário fechado, de autopreenchimento *on-line*. A análise estatística incluiu testes Qui-quadrado e modelos de regressão logística. A variável desfecho foi a presença de programa de prevenção ao uso de drogas inserido no cotidiano e no programa pedagógico da escola. As variáveis explicativas foram divididas em: dados demográficos do dirigente; características da escola e do currículo; educação em saúde; e consumo de drogas na escola.

**RESULTADOS:**

Constatou-se que 42,5% (IC95% 36,1–49,1) das escolas avaliadas possuíam programa de prevenção ao uso de drogas. Com o modelo de regressão logística múltipla, observou-se que, a cada ano de atuação do dirigente na educação, a chance de a escola ter um programa aumentava em aproximadamente 4,0%. O fato de experimentar técnicas de ensino inovadoras também aumentou em cerca de seis vezes a chance de a escola desenvolver um programa de prevenção ao uso de drogas. As dificuldades na implantação dos programas foram mais presentes nas redes estadual e municipal, quando comparadas à rede privada, destacando-se: a falta de material didático, a falta de dinheiro e as demandas concorrentes para ensino de outras disciplinas.

**CONCLUSÕES:**

A implantação de programas de prevenção ao uso de drogas no município de São Paulo está associada à experiência do dirigente escolar na educação e nas estratégias de ensino da escola.

## INTRODUCTION

The abusive consumption of alcohol and other drugs is a problem among adolescents in several parts of the world^1^. In Brazil, population-based epidemiological studies conducted in recent years indicate that the beginning of consumption of alcohol, tobacco, and other drugs occurs predominantly during adolescence^12,13,21^, which makes it a serious social and public health problem. However, although actions for drug abuse prevention are needed during adolescence, they should happen preferably before this phase^8,14^.

Studies that evaluated the results of the various programs for drug abuse prevention for students show that some programs collaborate to a significant reduction in the consumption of drugs by increasing negative attitudes regarding this use and improving the students’ resistance skills^5^. However, not all prevention programs implemented in schools are effective^5^.

Although various international studies assess school programs for prevention, studies on factors associated with the decision of implementing these programs are less frequent^11^. In Brazil, a country with little tradition in the development of this type of school program, the prevalence of programs for drug abuse prevention is unknown. In addition, little is known about the factors that influence the adoption of programs for drug abuse prevention in these institutions and even less about their effectiveness.

In the United States, the prevalence of programs for drug abuse prevention in schools is 72.0%, of which only 35.0% use programs with scientific evidence^7^. In this country, factors associated with the adoption of a program with scientific evidence of efficacy or effectiveness observed that school managers are the main responsibles for making the decision of adopting a program of drug use prevention^17^. Prevention coordinators of education secretariats are influential in the selection of curricula with proven scientific evidence, playing an important role in supporting managers and facilitating decision-making in the adoption of a program^17^. Some characteristics of the organizational context seem to be associated with the decision by the implementation of evidence-based programs in schools, such as the greater time devoted by managers in the activities for drug abuse prevention in schools^15^ and the availability of financial resources, due to the high cost of teacher training and the purchase of materials to develop the program^10^. In addition, the interface between schools and government agencies for research funding seems to favor the implementation of programs in schools. In this sense, educational institutions that received informational materials and research findings on prevention from the National Institute on Drug Abuse were more prone to apply effective prevention programs for their students^16^.

In general, studies that assess potential facilitators and barriers to implement the programs allow a political-pedagogical decision making that focus on the future introduction of prevention programs in the school curriculum, in a partnership between the school and the governmental sectors involved in the management or supervision of these institutions^18^.

On the international scene, the current discussion focuses on the level of scientific evidence of the programs implemented in schools. In Brazil, the discussion is still arising, and the definition, on the part of schools, of prevention programs and distribution of these programs in public and private schools remain unknown.

Given that, this study aimed to analyze if characteristics of managers, schools, and curriculum are associated with the implementation of programs for drug abuse prevention in elementary and high schools.

## METHODS

In this population-based cross-sectional survey, a probabilistic sample of managers of public and private schools of the city of Sao Paulo was evaluated, based on a self-filling online questionnaire on the implementation of prevention programs in their schools. Here, we considered as managers of the school headmasters, pedagogical coordinators, or coordinators of the prevention programs (in cases in which this position existed).

The target population consisted of managers of public and private schools, composed of elementary (from the 6th grade) and high school classes of the city of Sao Paulo, Brazil. According to the survey Data Escola Brasil of the Instituto Nacional de Estudos e Pesquisas Educacionais Anísio Teixeira (INEP), the city of Sao Paulo, in 2011, had 2,268 elementary and high schools. Of these, 990 were private schools, 774 were state schools, and 534 municipal schools.

To estimate the sample, we considered: the finite universe of schools of the municipality (n = 2,268); a 95% confidence level; absolute error of 5.0%; and an expected answers ratio of 50.0%, which resulted in a sample of 329 schools.

For the probabilistic selection of schools, the systematic drawing technique was used, i.e., each school had equal chance of being chosen in the sampling process. The list of schools was ordered by Postal Code, and the sampling quota was determined by calculation of the systematic sampling technique 2,268/329 = 6.89 (k = N/n). The first school was chosen at random, and the others were drawn with an interval of seven to reach the total of 329 schools.

Data were collected from October 2012 to October 2013 using the internet. All managers were invited by message, sent to e-mail addresses of the schools. The program SurveyMonkey was used to send the e-mails, which allowed us to forward messages to the 329 e-mail addresses at the same time. Respondents who have not responded to the survey after several attempts were contacted by telephone, both for being invited verbally and to clarify possible doubts.

The tool SurveyMonkey is an electronic platform that allows the sending of online questionnaires and registration in a database, which then can be converted into an Excel® spreadsheet (https://pt.surveymonkey.com/).

The managers, after accessing the link available in the message, were sent to a screen with an informed consent form and, after reading it, chose to click on “accept” or “not accept”. Respondents who chose the option “not accept” were considered as denials, and those who agreed to participate were directed to the screen with the questions of the questionnaire. The consent form and ethical research procedures were approved by the Ethics Committee of Universidade Federal de São Paulo (Process 32,594).

A closed, self-filling, and anonymous questionnaire was applied via internet. Part of the questions were extracted from the questionnaire by Ringwalt et al.^19^ (2002) and others were created to respond to the need of understanding the characteristics of Brazilian programs, and tested concerning understanding in a pilot study.

The outcome variable considered in the analysis of logistic regression data was the presence of program for drug abuse prevention in the daily life and educational program of the school. The explanatory variables were divided into: demographic data of the manager; characteristics of the school and of the curriculum; and drug use in the school.

The demographic variables were: sex (female; male); age (up to 35 years old, 36-45 years old, 46-55 years old, and 56 years old or over); time working in the current position, in the school, and with education (in years); and participation in courses with the theme drugs (yes; no).

The characteristics of the schools were: level of education offered (only elementary school; elementary school + high school; and only high school); type of school (state, municipal, and private); and size of the school (set from the total of students of all levels). We considered as small, schools with up to 800 students; medium sized, schools with 801–1,600 students; and large, schools with more than 1,600 students.

As for the school curriculum, we evaluated whether the school experimented with innovations (“our school often experiments with innovative curricula, programs, and educational practices”), evaluated into two categories (yes; no), from the grouping of replies: I totally agree and I partially agree, and I totally disagree, I partially disagree and indifferent (no); if the school develops activities to approach topics related to sexuality (yes; no); if the school develops activities to approach eating habits (yes; no).

The perception of drug use in schools was assessed by binary response variables (yes; no) regarding catching students carrying or consuming: 1) illicit drugs; 2) alcohol; and 3) tobacco.

In the descriptive analysis, quantitative variables were presented according to medians and interquartile intervals or absolute and relative frequencies and their respective 95% confidence intervals. With the aim of identifying factors associated with the implementation of programs for drug abuse prevention, logistic regression models were adjusted, considering as dependent variable (outcome) the presence of the prevention program. The independent variables (explanatory) analyzed were those related to the profile of the managers, characteristics of schools, subjects taught, developed activities, and history of catching students using drugs. Initially, we analyzed the relationship between each explanatory variable and the dependent variable for logistic regression models (univariate approach). The variables that presented p ≤ 0.15 were selected for a multivariate model, in which the effects were simultaneously analyzed. From an initial model with the selected variables, the variables without statistical significance were excluded step by step until a final model was obtained, with only the significant variables. The level of significance for hypothesis tests and for the final model was 5.0%. All analyses were carried out using Stata 12 program.

## RESULTS

Of the 329 schools selected and contacted, 279 (84.8%) agreed to participate in the study. However, 16 (5.7%) were excluded for having responded less than 30.0% of the questionnaire.

Characteristics of schools and respondent managers are presented in Table 1. In general, the schools that participated in this study offered from two to three levels of education, but municipal schools offered only elementary school. Most schools were public (state or municipal). Of the 263 managers who participated in the study, 52.5% were pedagogic coordinators, 44.5% were headmasters, and 3.0% were coordinators of programs for drug abuse prevention. There was a predominance of female respondents (76.4%) and aged between 36 and 55 years (73.4%). Regarding schooling, 64.2% had completed some post-graduate course (specialization, master’s, or PhD). Working time on the current school ranged from less than a year to 36 years (median: five years). Over half of respondents had at least 20 years of experience in the area of education and more than four years in the current position. Most managers had participated in training courses on drugs, most of these with course load time over eight hours.

**Table 1:** Characteristics of schools and respondent managers. Sao Paulo, SP, Southeastern Brazil, 2013. (N = 263)

Characteristics	n	%	95%CI
Characteristics of the school			
Level of education offered			
Only Elementary School	113	43.0	36.9–49.2
Elementary and High School	135	51.3	45.1–57.5
Only High School	15	5.7	5.1–6.3
Type of school			
State	111	42.2	36.2–48.4
Municipal	93	35.4	29.6–41.5
Private	59	22.4	17.5–28.0
Size			
Small (up to 800 students)	99	37.6	31.8–43.8
Medium (801-1,600 students)	126	47.9	41.7–54.1
Large (≥ 1,600 students)	38	14.5	10.4–19.3
Characteristics of the managers			
Sex			
Female	201	76.4	70.8–81.4
Male	62	23.6	18.5–29.2
Age group (years)			
≤ 35	45	17.1	12.7–22.2
36-45	96	36.5	30.7–42.6
46-55	97	36.9	31.0–43.0
≥ 56	25	9.5	9.1–9.7
Education			
High school	2	0.8	7.4–8.4
Higher education	92	34.9	29.2–41.1
Post-graduation (specialization)	136	51.7	45.5–57.9
Post-graduation (master’s or PhD)	33	12.5	8.8–17.2
Position at school			
Headmaster	117	44.5	38.4–50.7
Pedagogical coordinator	138	52.5	46.2–58.6
Prevention program coordinator	8	3.0	2.4–3.6
Working time (years)	Median		p25-p75
In the current position	4	-	2–8
In the current school	5	-	2–12
In the field of education	20	-	14–26
Participation of managers in courses on drugs*			
Yes	164	64.1	57.8–69.9
No	92	35.9	30.0–42.1

* 3.0% did not respond.

Most participants (94.8%) considered that the schools’ managers were open to changes. We also noted that 85.4% experimented with curriculum, programs, and teaching practices that they considered innovative. Regarding the activities developed in the schools, most of them, regardless of the type, worked contents related to health, sexuality, and eating habits.

Almost 80.0% of schools had already caught students using or carrying drugs, licit or illicit, in their establishments (Table 2). Tobacco was the drug most caught among students (69.9%). However, more than half of schools had already caught students using or carrying illegal drugs.

**Table 2:** Health education and drugs in the school context. Sao Paulo, SP, Southeastern Brazil, 2013. (N = 263)

Barriers	Total		State		Municipal		Private		p
			
	(n = 263)		(n = 111)		(n = 93)		(n = 59)		
			
	n (%)	95%CI	n (%)	95%CI	n (%)	95%CI	n (%)	95%CI	
Has a prevention program inserted in the daily life and educational program of the school	100 (42.5)	36.1–49.1	39 (39.4)	29.7–49.7	36 (42.4)	31.7–53.5	25 (49.0)	34.7–63.4	0.528
Managers open to changes	219 (94.8)	91.10–97.3	91 (94.8)	88.3–98.3	79 (94.0)	86.6–98.0	49 (96.0)	86.5–99.5	0.876
Experiment with innovative curricula, programs, and teaching practices	200 (85.8)	80.7–90.0	83 (84.7)	76.0–91.2	72 (85.7)	76.4–92.4	45 (88.2)	76.1–95.6	0.840
Develops activities to work with content linked to sexuality	228 (86.7)	82.0–90.5	97 (87.4)	79.7–92.9	78 (83.9)	74.8–90.7	53 (89.8)	79.2–96.2	0.140
Develops activities to work with content linked to eating habits	236 (89.7)	85.4–93.1	97 (87.4)	79.7–92.9	82 (88.2)	79.8–93.9	57 (96.6)	88.3–99.6	0.551
Caught students using illicit drugs inside the school	145 (56.6)	50.3–62.8	75 (68.8)^a,b^	59.2–77.3	46 (50.0)	39.4–60.6	24 (43.6)	30.3–57.7	0.002
Caught students using alcohol inside the school	149 (58.2)	51.9–64.3	76 (69.7)^a,b^	60.2–78.2	48 (52.2)	41.5–62.7	25 (45.5)	32.0–59.4	0.004
Caught students using tobacco inside the school	179 (69.9)	63.9–75.5	88 (80.7)^a,b^	72.1–87.7	58 (63.0)	52.3–72.9	33 (60.0)	45.9–73.0	0.005

^a^ Statistically significant difference when compared with municipal schools.

^b^ Statistically significant difference when compared with private schools.

Table 3 presents the barriers that hinder the implementation of programs for drug abuse prevention in schools. These difficulties in the implementation of the programs were more present in state and municipal schools, when compared with private schools, especially due to lack of financial investments. It is possible to highlight: teaching materials, lack of money, and competing demands for teaching other subjects.

**Table 3:** Barriers that hinder the implementation of programs for drug abuse prevention in schools. Sao Paulo, SP, Southeastern Brazil, 2013. (N = 263)

Barriers	Total		State		Municipal		Private		p
			
	(n = 235)		(n = 99)		(n = 85)		(n = 51)		
			
	n (%)	95%CI	n (%)	95%CI	n (%)	95%CI	n (%)	95%CI	
Lack of time for training teachers	165 (71.1)	64.8–76.8	73 (73.7)	63.9–82.1	63 (74.1)	63.5–83.0	29 (60.4)	46.1–74.2	0.185
Lack of appropriate material	148 (64.6)	58.2–70.8	73 (75.3)^b^	66.1–83.8	59 (71.1)^b^	59.7–80.0	16 (32.7)	20.8–47.9	< 0.001
Competing demands for teaching other subjects	136 (59.1)	52.6–65.5	49 (51.0)^a^	40.3–60.7	59 (71.1)	59.7–80.0	28 (54.9)	40.3–68.9	0.019
Lack of money	109 (47.6)	41.1–54.2	62 (63.3)^a,b^	53.4–73.1	37 (44.6)^b^	33.9–55.9	10 (20.8)	11.3–35.3	< 0.001
It is not high priority for teachers	63 (27.7)	22.0–33.8	24 (24.7)	16.2–33.9	26 (32.1)	22.1–42.8	13 (26.5)	15.9–41.7	0.538
Lack of support from teachers	53 (23.2)	18.1–29.3	27 (27.6)^b^	18.8–37.1	23 (28.4)^b^	19.0–39.0	3 (6.1)	4.6–7.4	0.006
It is not high priority for headmasters	45 (19.6)	14.7–25.2	20 (20.4)	12.8–29.5	15 (18.3)	11.1–28.8	10 (20.0)	9.8–33.1	0.935
Traffic pressure	43 (18.7)	13.9–24.3	21 (21.2)^b^	13.6–30.6	21 (25.6)^b^	17.0–36.5	1 (2.0)	0.9–3.3	0.003
Resistance of the school board and parents	29 (12.6)	8.8–17.7	14 (14.4)	7.9–22.6	9 (10.8)	5.0–19.1	6 (12.0)	4.4–23.9	0.762

^a^ Statistically significant difference when compared with municipal schools.

^b^ Statistically significant difference when compared with private schools.

Table 4 presents the factors associated with the implementation of prevention programs in schools. The chance of developing programs for drug abuse prevention was higher among schools with managers who had been working in the area of education for a longer time. According to the odds ratio point estimation, every year that the manager has worked with education increased the chance of the school having a program at about 4.0%. Experimenting with innovative teaching techniques also significantly increased the chance of the school inserting a program for drug abuse prevention in its daily life or education program. Working with activities related to the theme of sexuality presented a significant trend (p = 0.056), suggesting that schools which worked with sexuality tended to have more prevention programs than those that did not address this theme, though the difference was not statistically significant.

**Table 4:** Factors associated with the implementation of program for drug abuse prevention – results of univariate analysis and final multivariate model. Sao Paulo, SP, Southeastern Brazil, 2013. (N = 263)

Variable	Univariate			Multivariate		
	
	OR	95%CI	p	OR	95%CI	p
Characteristics of the managers						
Age (years)	1.02	0.99–1.05	0.182	-	-	-
Time in education (years)	1.03	1.00–1.07	0.044	1.042	1.01–1.08	0.023
Time at the school (years)	1.03	0.99–1.06	0.121	-	-	-
Time in the position (years)	1.05	1.00–1.09	0.047	-	-	-
Attended a prevention course	1.08	0.62–1.88	0.796	-	-	-
Characteristics of the school						
Level of education offered						
Elementary School	1.00	-	-	-	-	-
Elementary and High School	1.09	0.64–1.85	0.759	-	-	-
High School	0.40	0.10–1.56	0.189	-	-	-
Type of school						
State	1.00	-	-	-	-	-
Municipal	0.99	0.58–1.69	0.963	1.14	0.61–2.13	0.689
Private	1.40	0.75–2.60	0.292	1.18	0.57–2.47	0.652
Size of the school						
Small	1.13	0.49–2.57	0.777	-	-	-
Medium	1.07	0.49–2.38	0.859	-	-	-
Large	1.00	-	-	-	-	-
Managers open to changes^a^	1.53	0.45–5.52	0.496	-	-	-
Experiment with innovative curricula, programs, and teaching practices^b^	6.69	2.27–19.74	0.001	6.19	2.01–19.04	0.001
Health and drugs education in the school context						
Develops activities to work with content linked to sexuality	4.928	1.65–14.75	0.004	3.11	0.971–9.95	0.056
Develops activities to work with content linked to eating habits	2.257	0.86–5.95	0.100	-	-	-
Caught students using illicit drugs inside the school	0.99	0.59–1.66	0.964	-	-	-
Caught students using alcohol inside the school	0.73	0.43–1.24	0.251	-	-	-
Caught students using tobacco inside the school	0.89	0.51–1.56	0.697	-	-	-

^a^ Fully or partially agreed with the statement “the managers in our school are open to change”.

^b^ Fully or partly agreed with the statement “our school often experiments with innovative curricula, programs, and education practices”.

## DISCUSSION

Less than half of the elementary and high schools studied have a program of drug abuse prevention inserted into the curriculum. The factors associated with the implementation of programs in the school were: headmaster’s experience in education, declaring to experiment with innovative curricula, and also having programs on sexuality.

Although the school is considered by society as a protective environment, this study showed a scenario that deserves attention, with high level of flagrant consumption and possession of drugs inside the schools, mainly in public ones. These data corroborate the students’ self-report on consumption, obtained in an epidemiological survey performed in the year of 2010 among students of the city of Sao Paulo. In this survey, the prevalence of drugs use (except alcohol and tobacco) was 23.3% for use in life, 9.6% for use in the year, and 5.2% for use in the month. The prevalence of alcohol consumption was highest, being 60.4%, 42.9%, and 20.7% for use in life, in the year and in the month, respectively^3^.

Schools whose managers had more time working in the area of education presented a higher chance to implement a program for drug abuse prevention. This result can be associated with the insecurity of the managers who work less time in the area of education. In fact, a study performed in schools of the municipality of Sao Paulo showed that pedagogical coordinators feel unprepared to develop a program for drug abuse prevention in their schools^2^. In addition, managers more experienced in education tend to know more about the social reality and the needs of students who attend their school^17^.

Data from this study suggest that schools that tested innovative teaching practices develop new curricula and more programs for drug abuse prevention than schools that did not test new practices. The term innovation represents a change in the existing practice, suggesting that schools that developed programs for drug abuse prevention were more open to changes and transformations in the school context. For Ferretti^6^ (1995), to innovate the curriculum means to propose diversified activities to integrate the various aspects of the development of the student. This result suggests that these programs are offering content based on demands of the schools and the students themselves, making the teaching-learning process more active.

We also observed an association between the practice of prevention curriculum and activities on sexual education, suggesting that schools that developed programs for drug abuse prevention were simultaneously working with more than one risk behavior and potentially integrating the information about drug abuse with information about sexual behavior. Indeed, programs that integrate other risk behaviors to the theme of drugs tend to show greater effectiveness in prevention than programs that separately deal with the question of drug use among students^9,20^.

The results suggest that the processes of daily life, such as lack of time for teacher training and competing demands for other subjects, are challenges to the implementation of programs for drug abuse prevention in schools of Sao Paulo, indicating the need for adjustments in the structure of the school curriculum and routine.

Economic restrictions emerged as the greatest challenge in the implementation process, especially in public schools, in which we observed a lack of money and of suitable material for the development of programs for drug abuse prevention, which shows the need for investments in prevention in schools of Sao Paulo. Various prevention programs, with effectiveness detected by randomized controlled trials^5^, do not require big economic investment, and are an option especially for schools with economic barriers to the implementation of programs. However, investment in the training of school managers is essential, allowing access to several programs, among which they could choose the one that best adapt to their school community^4^. This type of measure should be established from public policies in the sector of education, allowing the creation of a preventive culture between headmasters and teachers of schools in Brazil, as isolated actions can hardly cover the complexity of the theme and change of established routines.

The targeting of new preventive practices in the school context is only possible with more investments from the public and private sectors. Consistent alliances between health and education sectors can also contribute, as long as they are aimed at the integration and permanent linking of these sectors for the development of programs for drug abuse prevention.

Despite being the first study that assessed the prevalence and characteristics of the programs by probabilistic sample in the city of Sao Paulo, there are some limitations. With the isolated use of quantitative methodology, we were unable to identify if the questions were precisely understood by respondents and whether the programs implemented were based on evidence of results. Regarding the analyses, some estimates of the logistic regression models were inaccurate, which is evidenced by the extent of the confidence intervals for the odds ratio, explained by the low frequency of schools with negative responses to some questions. Finally, we did not evaluate the quality of the programs implemented, since this is not an effectiveness evaluation study. Thus, future studies should be designed to evaluate the efficacy and effectiveness of these programs, contributing to decide the best program regarding cost-effectiveness.
